# Ross-Kamm

**Published:** 1853-08

**Authors:** 


*Ross-Kamm.—Notice Gratis!*—A reader of the smart efforts at puff furnished by the local of the Buffalo Daily Express, repeated there *ad nauseam*, week by week, has asked what means this thin-skin lionizing of a Dr. Ross, as "*the* eye and ear Doctor," somewhat recently come among us? The name and use of it in that print is all that we have suggestive of answer.

Our caption, from the German, in our vernacular may be rendered "Jockey" perhaps more freely "Ross-came." That he "came, saw and conquered," we have not learned outside of the Express. That this subject of puff has been often "trotted out," all know. That he has had a "run" (unless this notice shall be so construed, or he is now with those of like ilk, "o'er the hills and far away,") we are not clear. Perhaps it is "all in the eye," to the puffee, and surely it adds "more nor leetle" to the elongated "ear" of the puffer. He undoubtedly has undertaken to "stand for the season," as a mule generator, mayhap Centaur, from the close embrace of the parties. That this "Ross" has made many moves since, here, that veracious and well-informed out-rider has attested; and, lastly, (for it is the last if he puffs not again while we write,) regrets that this "hobby-horse" threatens to leave for Philadelphia. The ☞ points now to a broken-winded issue, a *sorry-horse*, oft belabored, but one, unless new jockeyed, that on this course is no go.

The liberty taken above in the turn of a name, may have the appearance of grossness to the readers of this Journal. In palliation, we add, we have recently turned from the Express where even the advertised Post-office list furnishes its real names for such overturning—the owners of them unprotected by quill or paper for responses, while the unrebuked puffing at the name above, shows where there is a liking to it. We for once, while in Rome, dealing with Romans, have concluded to do as Romans do.

